# Hypervascular solid-appearing serous cystadenoma as a mimic of nonfunctioning pancreatic neuroendocrine tumour: a systematic review

**DOI:** 10.1007/s00261-026-05532-y

**Published:** 2026-04-25

**Authors:** Mario Annecchiarico, Giuseppe Loiaco, Giulio Argenio, Antonio Varricchio, Gianluca Rompianesi, Roberto Cannella

**Affiliations:** 1 General and Oncological Surgery Unit, AORN “San Pio”, 82100 Benevento, Italy; 2 Clinical and Translational Oncology PhD Programme, Scuola Superiore Meridionale, Naples, Italy; 3 Department of Clinical Medicine and Surgery, Division of HBP, Minimally Invasive and Robotic Surgery, Transplantation Center, University of Naples Federico II, Naples, Italy; 4 Department of Biomedicine, Neuroscience and Advanced Diagnostics (BiND), University of Palermo, 90127 Palermo, Italy

**Keywords:** Serous cystic adenoma, Pancreatic neuroendocrine tumour, Differential diagnosis, Computed tomography, Magnetic resonance imaging, Somatostatin receptor imaging

## Abstract

**Background:**

Solid pancreatic serous cystadenoma (SCA) may present as a hypervascular mass and mimic nonfunctioning pancreatic neuroendocrine tumour (NF-pNET), potentially leading to pancreatic resection for benign disease. We synthesized the available evidence on this diagnostic pitfall.

**Methods:**

A PRISMA 2020–aligned systematic review searched MEDLINE, Embase, and Web of Science (1 January 2000–1 December 2025). We included comparative cohorts of pathology-proven SCA versus NF-pNET reporting CT/MRI discriminative features and case reports/series of pathology-proven SCA preoperatively interpreted as NF-pNET. Comparative cohorts and case reports/series were synthesized separately; no meta-analysis was performed.

**Results:**

Thirty-five publications were included: four retrospective single-center comparative cohorts (50 SCA and 89 NF-pNET patients) and 31 case reports/series (39 SCA patients). In comparative cohorts, unenhanced CT attenuation was consistently lower in SCA than in NF-pNET (18.5–29.6 vs. 38.8–48.4 HU), with study-specific ROC-derived cutoffs in the low 30-HU range; attenuation ratios performed similarly. Diffusion-weighted MRI showed higher ADC values in SCA, with reported study-specific cutoffs of > 1.36 × 10^− 3^ mm^2^/s and > 1.99 × 10^− 3^ mm^2^/s. In case reports/series, arterial hyperenhancement occurred in 32/33 (97.0%), ^68^Ga-DOTA–peptide uptake in 17/39 (43.6%), and EUS-guided sampling was diagnostic in 9/17 (52.9%).

**Conclusions:**

NF-pNET–mimicking SCA is an uncommon but clinically consequential benign mimic. In the available retrospective comparative cohorts of resected, pathology-proven lesions, lower unenhanced CT attenuation (and attenuation ratios) and higher DWI–ADC values consistently favored SCA over NF-pNET, whereas arterial hyperenhancement and positive somatostatin receptor imaging uptake were not specific. These findings may support cautious multiparametric assessment in selected hypervascular solid-appearing pancreatic lesions, but should not be applied as definitive diagnostic thresholds without external validation.

**Supplementary Information:**

The online version contains supplementary material available at https://doi.org/10.1007/s00261-026-05532-y.

## Introduction

Pancreatic serous cystadenoma (SCA) is a relatively uncommon cystic tumour, accounting for approximately 1–2% of all pancreatic lesions and about 20% of pancreatic cystic neoplasms [1, 2]. Large multicentric cohort studies indicate an almost invariably benign course with negligible disease-specific mortality, supporting conservative management in asymptomatic patients [3]. In its classic microcystic variant, SCA appears on cross-sectional imaging as a well-circumscribed sponge-like lesion composed of numerous small cysts separated by thin septa, occasionally with a central fibrous scar or calcifications [4]. When these classic features are present, the non-invasive diagnosis is usually straightforward. However, SCA may lack the typical microcystic pattern and occasionally present as macrocystic/oligocystic, mixed, or even solid variants, thereby increasing the risk of misdiagnosis [5].

Solid SCA is a recognised radiological pitfall; it may present as a hypervascular solid lesion, thus mimicking potentially malignant pancreatic lesions, particularly nonfunctioning pancreatic neuroendocrine tumour (NF-pNET) [6]. The solid variant is rare, and diagnostic confusion typically arises when individual microcysts fall below the spatial resolution of conventional cross-sectional imaging (computed tomography [CT] or magnetic resonance imaging [MRI]), resulting in a pseudo-solid hypervascular appearance [7].

NF-pNET represents a biologically heterogeneous neoplasm with variable malignant potential; management is individualized and ranges from active surveillance for selected small, low-risk lesions to surgical resection for tumours with higher-risk features or symptomatic disease [8]. In routine practice, a well-circumscribed hypervascular pancreatic mass is often interpreted as NF-pNET, particularly when somatostatin receptor imaging (SRI) is concordant with CT or MRI findings [9, 10]. However, SRI tracer uptake is not exclusive to NF-pNET and has also been described in SCA [11–14]. In this setting, the diagnostic dilemma may persist despite endoscopic ultrasound (EUS)–guided tissue acquisition, which has shown low sensitivity for diagnosing SCA [15]. As a consequence, patients may ultimately undergo major pancreatic resections for lesions proved to be benign.

Evidence on SCA interpreted as NF-pNET is scattered across small cohorts and case reports/series. This systematic review synthesises the available evidence to characterise NF-pNET–mimicking SCA, identify potentially discriminative imaging features, and summarise downstream management and outcomes reported in this setting.

## Methods

### Protocol and review objectives

The study protocol was registered in PROSPERO (CRD420251181677) [16] and the review was conducted in accordance with PRISMA 2020 [17]. The diagnostic question was framed using a PIRD structure (population, index test, reference standard, diagnosis) [18]. The primary objective was to evaluate CT and MRI features reported to differentiate NF-pNET–mimicking SCA from true NF-pNET in comparative cohorts. Secondary objectives were to characterize diagnostic pathways leading to preoperative misinterpretation of SCA as NF-pNET in case reports/series, including the role of somatostatin receptor imaging (SRI) and endoscopic ultrasound (EUS), to summarize histopathological and immunohistochemical findings supporting the final diagnosis, and to describe management and postoperative outcomes.

Primary outcomes were cohort-reported CT/MRI discriminative features and study-level diagnostic performance metrics (e.g., ROC-derived thresholds and AUC for unenhanced attenuation, attenuation ratios, and apparent diffusion coefficient [ADC]). Secondary outcomes included the suspected preoperative diagnosis or differential diagnosis; use of EUS-guided tissue acquisition; SRI tracer type and SUVmax (when reported); and management outcomes (type of operation, complications, length of stay, and follow-up).

### Eligibility criteria

Studies were eligible if published between 1 January 2000 and 1 December 2025 (last search), available in full text in English, and involving adults (≥ 18 years). We included comparative cohort studies of pathology-proven SCA versus NF-pNET and case reports or series of pathology-proven SCA misinterpreted as NF-pNET or explicitly included in the differential diagnosis. A pathological reference standard (surgical histology or biopsy-based cytology/histology) and at least one imaging modality (CT, MRI, or SRI) were required.

SRI encompassed ^68^Ga-DOTA–peptide PET/CT or PET/MRI and Octreoscan scintigraphy, with or without SPECT/CT. When available, EUS findings and EUS-guided tissue acquisition were extracted. Exclusion criteria included pediatric populations, non-original articles, conference abstracts without full text, purely technical imaging studies, and mixed cystic cohorts or concomitant NF-pNET not reported separately.

Data extractability requirements differed by study design: patient-level data were required for case reports/series, whereas group-level data comparing SCA and NF-pNET were sufficient for comparative cohorts.

### Search strategy and study selection

MEDLINE, Embase, and Web of Science were searched from 1 January 2000 to 1 December 2025 using a combination of controlled vocabulary (MeSH/Emtree) and free-text terms related to pancreatic serous neoplasms (e.g., “serous cystadenoma,” “serous neoplasm,” “serous adenoma,” “solid serous”) and pancreatic neuroendocrine tumours (e.g., “neuroendocrine,” “NET,” “islet cell”). Search strategies were adapted for each database. Reference lists of included studies and relevant reviews were manually screened to identify additional records.

After deduplication, titles/abstracts and full texts were independently screened by two reviewers (GL and GA). Disagreements were resolved by consensus or, when necessary, adjudication by a third reviewer (MA).

### Data extraction, risk of bias and data synthesis

Data were independently extracted by two reviewers (GL and RC) using a piloted data-collection form. Discrepancies were resolved through discussion and, when necessary, adjudication by a third reviewer (MA).

For comparative cohort studies, data were extracted at the group level (SCA vs. NF-pNET), whereas case reports and series were extracted at the individual patient level. Prespecified variables included imaging features (CT, MRI, SRI) as well as relevant clinical, pathological, management, and outcome data when available. SCA subtypes were coded according to the Reid et al. classification when reported [4].

Risk of bias was assessed separately by study design. Comparative cohorts were appraised with QUADAS-2 [19], and case reports/series with the Murad et al. tool [20]. Risk of bias was independently assessed by two reviewers (GL and AV), with adjudication by a third reviewer (MA) when necessary. Judgments informed interpretation of the findings but did not determine study eligibility.

Comparative cohort studies and case reports/series were synthesized separately. Cohort studies provided group-level comparative metrics (SCA vs. NF-pNET), whereas case reports/series described individual diagnostic pathways. Quantitative pooling was not performed because of substantial clinical and methodological heterogeneity across cohorts, including differences in acquisition protocols, region-of-interest definitions, non-overlapping quantitative metrics, and ROC-derived thresholds not reported as extractable 2 × 2 data.

Cohort data were therefore summarized descriptively using counts and percentages or means with standard deviations, as reported. Case reports and series were synthesized narratively to characterize diagnostic pathways, imaging features, and management outcomes. When medians and ranges were reported, means and standard deviations were estimated using the method of Wan et al. to allow consistent descriptive reporting [21].

## Results

### Study selection and study characteristics

The literature search identified 1283 records. After title/abstract and full-text screening, 35 publications met the eligibility criteria and were included: four comparative cohort studies [22–25] and 31 case reports/series (27 case reports and 4 case series) [11–14, 26–52]. All comparative cohorts were retrospective single-center studies. The cohort studies provided group-level data comparing pathologically confirmed SCA and NF-pNET, whereas the case-based literature described pathologically confirmed SCA that was preoperatively interpreted as NF-pNET or in which NF-pNET was explicitly included in the differential diagnosis.

**Fig. 1:**
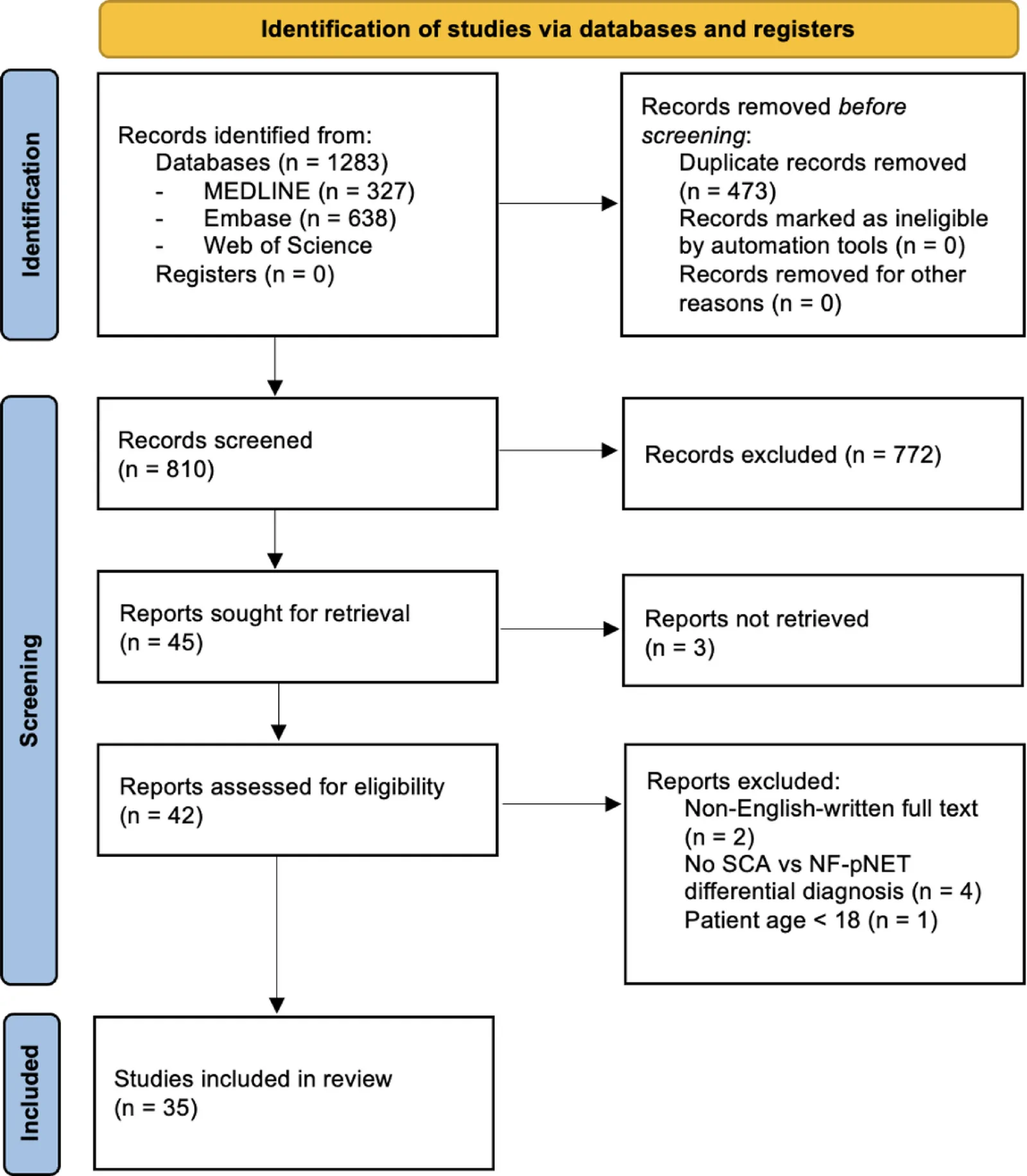
PRISMA 2020 flow diagram of study selection

Study selection is summarized in the PRISMA 2020 flow diagram (Fig. 1).

Key characteristics of the comparative cohorts and case reports/series are presented in supplementary materials (Tables S1 and S2), respectively.

### Study quality and risk of bias

Across comparative cohorts, patient selection represented the main methodological limitation, with all studies judged at high risk of bias and high applicability concern in this domain, reflecting retrospective resected-series sampling [22–25]. In contrast, the reference standard domain was consistently rated as low risk of bias and low concern regarding applicability across all cohorts. Ratings for the index test were high risk of bias in 2/4 studies and unclear in 2/4; for flow and timing, risk of bias was high in 2/4, unclear in 1/4 and low in 1/4.

In the case reports/series, overall methodological concern was rated as low in 4/31 reports, moderate in 26/31 and high in 1/31 [11–14, 26–52]. Full risk-of-bias assessments are provided in supplementary materials (Tables S3–S4).

### Baseline characteristics

The four comparative cohorts included 50 patients with SCA and 89 with NF-pNET. Lesion location was reported for all SCA cases and for 74/89 NF-pNET cases. Symptom status was available for 15/50 SCA and 30/89 NF-pNET patients [22–25]. The case reports/series contributed 39 patients with pathologically confirmed SCA, with symptom status available for 37/39 patients [11–14, 26–31, 33–51]. Clinical characteristics are summarized in Table 1.

**Table 1:** Baseline characteristics in comparative cohorts and case reports/series

Characteristic	Comparative cohorts		Case-based literature
	SCA (*n* = 50)	NF-pNET (*n* = 89)	SCA (*n* = 39)
Age, years (mean ± SD)	60.7 ± 10.5	54.9 ± 11.6	60.85 ± 10.99
Female sex, n/N (%)	34/50 (68.0)	51/89 (57.3)	23/39 (59.0)
Location: head, n/N (%)	18/50 (36.0)	37/74 (50.0)	16/39 (41.0)
Location: neck, n/N (%)	2/50 (4.0)	2/74 (2.7)	4/39 (10.3)
Location: body–tail, n/N (%)	30/50 (60.0)	35/74 (47.3)	19/39 (48.7)
Lesion size, mm (mean ± SD)	25.2 ± 12.0	24.4 ± 14.1	32.25 ± 18.48
Symptomatic, n/N (%)	5/15 (33.3)	7/30 (23.3)	18/37 (48.6)
Abdominal pain, n/N (%)	–	–	13/18 (72.2)
Dyspeptic symptoms, n/N (%)	–	–	3/18 (16.7)
Weight loss, n/N (%)	–	–	2/18 (11.1)
Incidentally detected, n/N (%)	10/15 (66.6)	23/30 (76.6)	19/37 (51.4)

NF-pNET, nonfunctioning pancreatic neuroendocrine tumour; SCA, serous cystadenoma; SD, standard deviation

Denominators vary by feature reporting (NF-pNET location: 74/89; symptom status: cohorts 15 SCA and 30 NF-pNET; case-based 37/39)

### Imaging findings in comparative cohort studies

The four retrospective single-center comparative cohort studies reported imaging findings in patients with histologically confirmed SCA and NF-pNET [22–25]. The technical imaging parameters, ROI methodologies, and reported diagnostic thresholds of the four comparative cohort studies are detailed in Supplementary Table S5.

Multiphasic contrast-enhanced CT was available in all comparative cohorts. MRI was reported in two cohorts [22, 24], whereas comparative cohort data on SRI were not available. In these cohorts, unenhanced CT attenuation was consistently lower in SCA than in NF-pNET, with mean values ranging from 18.5 to 29.6 HU for SCA and from 38.8 to 48.4 HU for NF-pNET [22, 24, 25]. Two cohorts reported ROC-derived unenhanced attenuation cutoffs in the low 30-HU range. Park et al. reported a threshold of < 34 HU with a sensitivity of 73.3% and specificity of 73.3% [22]. Jang et al. reported a threshold of < 32 HU with an AUC of 0.973, a sensitivity of 100% and a specificity of 86.7% [24]. Park et al. reported a mean arterial-phase attenuation of 142.4 ± 35.6 HU in SCA and 146.5 ± 46.9 HU in NF-pNET, with no significant difference (*p* = 0.76) [22]. Fang et al. additionally evaluated lesion-to-pancreas attenuation value ratios (AVR) and an enhancement-pattern index. The unenhanced attenuation ratio performed best, with an unenhanced AVR threshold of < 0.77 yielding an AUC of 0.96, a sensitivity of 90.9% and a specificity of 86.2%, whereas arterial-phase AVR showed lower discrimination, with an AUC of 0.72 [23]. Detailed CT quantitative values and study-level performance metrics are reported in Tables 2 and 3.

**Table 2:** CT-derived thresholds and diagnostic performance for differentiating SCA from NF-pNET in comparative cohorts

First Author	Year	Index variable	Optimal cutoff	AUC	Sensitivity	Specificity
Jang et al.	2015	Unenhanced attenuation	< 32 HU	0.973 (0.817–1.000)	100%	86.7%
Park et al.	2016	Unenhanced attenuation	< 34 HU	NR	73.3%	73.3%
Fang et al.	2022	Unenhanced AVR	< 0.77	0.96 (0.92–1.00)	90.91%	86.24%
		Arterial AVR	< 1.08	0.72 (0.68–0.87)	63.64%	75.86%
		Portal AVR	< 0.99	0.80 (0.75–0.92)	72.73%	82.76%
		Delayed AVR	< 0.95	0.85 (0.75–0.97)	72.73%	93.10%
		Wash-in/wash-out	–	0.86 (0.76–0.98)	96.55%	77.27%

CT, computed tomography; HU, Hounsfield units; AVR, attenuation value ratio (lesion-to-pancreas); AUC, area under the ROC curve; NR, not reported

cutoffs are reported in the direction favouring SCA

**Table 3:** CT attenuation values reported in comparative cohort studies

First Author	Year	CT phase	SCA (mean ± SD; *n*)	NF-pNET (mean ± SD; *n*)	*p*-value*
Hayashi et al.	2012	Unenhanced	28.0 ± 8.5 HU (*n* = 2)	48.4 ± 11.2 HU (*n* = 11)	*p* < 0.05
		Arterial	188.5 ± 77.0 HU (*n* = 2)	181.1 ± 55.0 HU (*n* = 12)	NR
		Delayed	82.5 ± 27.6 HU (*n* = 2)	126.2 ± 35.3 HU (*n* = 12)	*p* = 0.14
Jang et al.	2015	Unenhanced	18.5 ± 8.16 HU (*n* = 10)	39.2 ± 7.76 HU (*n* = 15)	*p* < 0.001
Park et al.	2016	Unenhanced	29.6 ± 9.5 HU (*n* = 15)	38.8 ± 9.9 HU (*n* = 30)	*p* < 0.01
		Arterial	142.4 ± 35.6 HU (*n* = 15)	146.5 ± 46.9 HU (*n* = 30)	*p* = 0.76
		Portal venous	136.0 ± 34.3 HU (*n* = 15)	154.9 ± 44.8 HU (*n* = 30)	*p* = 0.15

HU, Hounsfield units; SD, standard deviation; NR, not reported; *p-values as reported by the original studies

In the two cohorts reporting diffusion-weighted imaging, ADC values were higher in SCA than in NF-pNET. Park et al. reported mean ADC values of 1.621 ± 0.139 × 10^− 3^ mm^2^/s in SCA and 1.056 ± 0.303 × 10^− 3^ mm^2^/s in NF-pNET (*p* < 0.01) [22]. Jang et al. reported mean ADC values of 2.45 ± 0.27 × 10^− 3^ mm^2^/s in SCA and 1.51 ± 0.46 × 10^− 3^ mm^2^/s in NF-pNET (*p* < 0.001) [24]. ROC-derived ADC thresholds were also reported, with study-specific cutoffs of > 1.36 × 10^− 3^ mm^2^/s and > 1.99 × 10^− 3^ mm^2^/s and high study-level discrimination [22, 24].

Comparative EUS data were reported in a single cohort [22]. EUS was performed in 7/15 patients with hypervascular solid-appearing SCA (46.7%) and in 16/30 patients with NF-pNET (53.3%). The EUS-based presumptive diagnosis matched final pathology in 3/7 SCA cases (42.9%) and 13/16 NF-pNET cases (81.3%). EUS-guided FNA was performed in 5/7 SCA and 10/16 NF-pNET patients undergoing EUS. Diagnostic material was obtained in 0/5 SCA cases (0%) and 6/10 NF-pNET cases (60%).

### Imaging findings in case reports/series

Across 39 case reports/series of SCA in which NF-pNET was the presumptive diagnosis or explicitly included in the differential diagnosis, CT was reported in 35/39 cases (89.7%) [11–14, 26–34, 36–40, 42–44, 47–52], MRI in 19/39 (48.7%) [11–13, 26, 28, 33, 35, 38, 40, 45, 46, 50], SRI in 17/39 (43.6%) [11–14, 34, 38, 39, 41–43, 46, 48, 51], and EUS in 17/39 (43.6%) [11, 13, 30, 36, 38, 40, 41, 46, 48, 51].

On CT, the arterial phase was explicitly described in 33 cases and demonstrated arterial hyperenhancement in 32/33 (97.0%). Portal or delayed phase characteristics were reported in 12 cases, with washout in 7/12 (58.3%), persistent enhancement in 4/12 (33.3%), and an indeterminate pattern in 1/12 (8.3%).

Qualitative unenhanced attenuation was described in 11 cases and was low density in 9/11 (81.8%) and isoattenuating in 2/11 (18.2%). Main pancreatic duct dilatation was assessed in 15 cases and was present in 3 (20.0%). Intralesional calcifications were evaluated in 7 cases and identified in 2 (28.6%). A central scar was reported in 1 of 6 cases in which this feature was specifically assessed (16.7%).

On MRI, T2 signal intensity was described in 15 cases and was high in 12 (80.0%) and very high in 3 (20.0%). T1 signal intensity was assessed in 7 cases and was low in all 7 (100%).

SRI modalities included ^68^Ga-DOTATOC PET/CT (*n* = 4) and ^68^Ga-DOTATATE PET/CT (*n* = 8); additional techniques comprised ^111^In-pentetreotide SPECT (*n* = 3), ^99m^Tc-HYNIC-TOC SPECT (*n* = 1), and ^18^F-FDG PET/CT (*n* = 1). Among the subset of reports providing SUVmax data (*n* = 7), the mean SUVmax was 11.2 ± 3.4 for ^68^Ga-DOTATOC (*n* = 4) and 9.0 ± 2.6 for ^68^Ga-DOTATATE (*n* = 3).

Among EUS reports, contrast-enhanced EUS was described in 8 cases and demonstrated hyperenhancement in 7 (87.5%) and hypoenhancement in 1 (12.5%). EUS-guided tissue acquisition was reported in 17 cases, yielding a diagnostic or adequate result in 9 (52.9%) and a non-diagnostic or insufficient result in 8 (47.1%).

### Management, outcomes and follow-up

In the comparative cohort studies, all included patients underwent pancreatic resection and had histopathological confirmation on resection specimens, reflecting retrospective resected-series sampling and the requirement for a pathological reference standard; however, none of the studies reported the type of pancreatic operation separately for SCA and NF-pNET [22–25]. In the series by Jang et al., an overall distribution was provided for the 25 operated patients (SCA and NF-pNET combined), with 18 undergoing partial pancreatectomy (72.0%) and 7 undergoing pancreatoduodenectomy (28.0%) [24].

In the case reports/series, surgery was performed in 30/39 cases (76.9%), while 9/39 (23.1%) were managed conservatively. Among operated patients, pancreatoduodenectomy was reported in 13 (43.3%), distal pancreatectomy in 14 (46.7%), and enucleation in 3 (10.0%). Among distal pancreatectomies (*n* = 14), splenectomy status was reported for 12 cases (85.7%), with splenectomy performed in 9 (75.0%). Postoperative complication status was reported in 16 cases (53.3%) and described in 3 (18.8%) (diarrhoea, biochemical leak, and grade B postoperative pancreatic fistula). Where complication severity was explicitly reported, no Clavien–Dindo grade ≥ IIIa event was documented. Postoperative length of stay, available only for 11 cases (36.7%), was 9.5 ± 4.3 days. Follow-up was reported in 23/39 cases (59.0%), with a mean duration of 18.2 ± 17.0 months. No recurrences were reported among the 11 cases that explicitly stated recurrence status.

### Histopathology and immunohistochemistry

Across the 89 patients with SCA included in the review, histopathological confirmation was obtained from resection specimens in all comparative cohort studies [22–25] and in 30 (76.9%) case reports/series [11–14, 26–40, 42–52], whereas the remaining 9 cases (23.1%) were confirmed by biopsy-based cytology or histology [41, 48]. Subtype distribution is summarized in Table 4. Subtype was specified for 79 patients (88.8%). Within the overall SCA cohort (*n* = 89), 67 (75.3%) were reported as solid and 12 (13.5%) as microcystic, while subtype was not specified in 10 (11.2%).

**Table 4:** Histological subtype distribution of NF-pNET-mimicking SCA across comparative cohorts and case-based reports

Histological subtype	Comparative cohorts (*n* = 50)	Case-based literature (*n* = 39)	Overall (*n* = 89)
Solid	44 (88.0%)	23 (59.0%)	67 (75.3%)
Microcystic	6 (12.0%)	6 (15.4%)	12 (13.5%)
Macrocystic/oligocystic	0 (0.0%)	0 (0.0%)	0 (0.0%)
Mixed	0 (0.0%)	0 (0.0%)	0 (0.0%)
Not reported	0 (0.0%)	10 (25.6%)	10 (11.2%)

SCA, serous cystadenoma; NF-pNET, nonfunctioning pancreatic neuroendocrine tumour

Immunohistochemistry, available exclusively in case reports/series (*n* = 22), demonstrated PAS positivity in all assessed cases. Epithelial markers were consistently expressed, with CK7 positive in 12/12 tested cases, CK19 in 7/7, AE1/AE3 in 5/5, CAM5.2 in 4/4, and EMA in 4/5. Markers supporting serous differentiation were positive when evaluated, with α-inhibin positive in 8/9 and MUC6 in 3/3. In contrast, neuroendocrine markers were uniformly negative: synaptophysin in 21/21 and chromogranin A in 12/12 assessed cases.

## Discussion

This systematic review highlights that SCA may occasionally mimic NF-pNET on preoperative assessment and that enhancement behavior alone is insufficient to resolve the differential diagnosis. When uncertainty persists in a resectable hypervascular solid pancreatic lesion, management may escalate to pancreatic resection for what ultimately proves to be a benign disease. This is particularly relevant because guideline frameworks support conservative management when a serous cystic neoplasm is confidently diagnosed, whereas surgical resection remains an accepted option for selected NF-pNET within guideline-based pathways [3, 8]. In our synthesis, most NF-pNET–mimicking SCA were reported as the solid variant (67/89, 75.3%), although microcystic lesions were also represented (12/89, 13.5%). This reflects the recognized spectrum of serous cystic neoplasms, in which small cystic spaces and enhancing septa may produce an apparently solid hypervascular mass [4, 53].

Comparative imaging findings help explain why morphology and hypervascularity are unreliable in isolation. Baseline quantitative CT and MRI measures may better reflect the fluid-rich serous architecture, even when the lesion appears solid. Across comparative cohorts, unenhanced CT attenuation was consistently lower in SCA than in NF-pNET (18.5–29.6 HU vs. 38.8–48.4 HU) [22, 24, 25]. Unenhanced attenuation and attenuation ratios showed the largest separation and the highest reported study-level discrimination among available CT metrics. Diffusion-weighted MRI showed concordant findings in the two cohorts reporting DWI, with higher ADC values in SCA than in NF-pNET and study-specific ROC-derived cutoffs of > 1.36 × 10^− 3^ mm^2^/s and > 1.99 × 10^− 3^ mm^2^/s [22, 24]. However, these data derive from only four retrospective single-center comparative cohorts composed of resected, pathology-proven lesions and therefore represent highly selected surgical populations. Accordingly, the reported ROC-derived thresholds should be interpreted as study-level, hypothesis-generating cutoffs rather than universally applicable decision thresholds. In addition, interstudy differences in CT acquisition, MRI technique, diffusion parameters, and ROI methodology may have influenced the reported thresholds and limit direct transferability to routine practice.

Functional imaging may further contribute to diagnostic ambiguity. Published cases document moderate to marked ^68^Ga-DOTA–peptide uptake in SCA, including microcystic lesions, indicating that a positive SRI signal does not exclude a benign serous diagnosis and should be interpreted alongside cross-sectional imaging findings and, when available, tissue results.

**Fig. 2:**
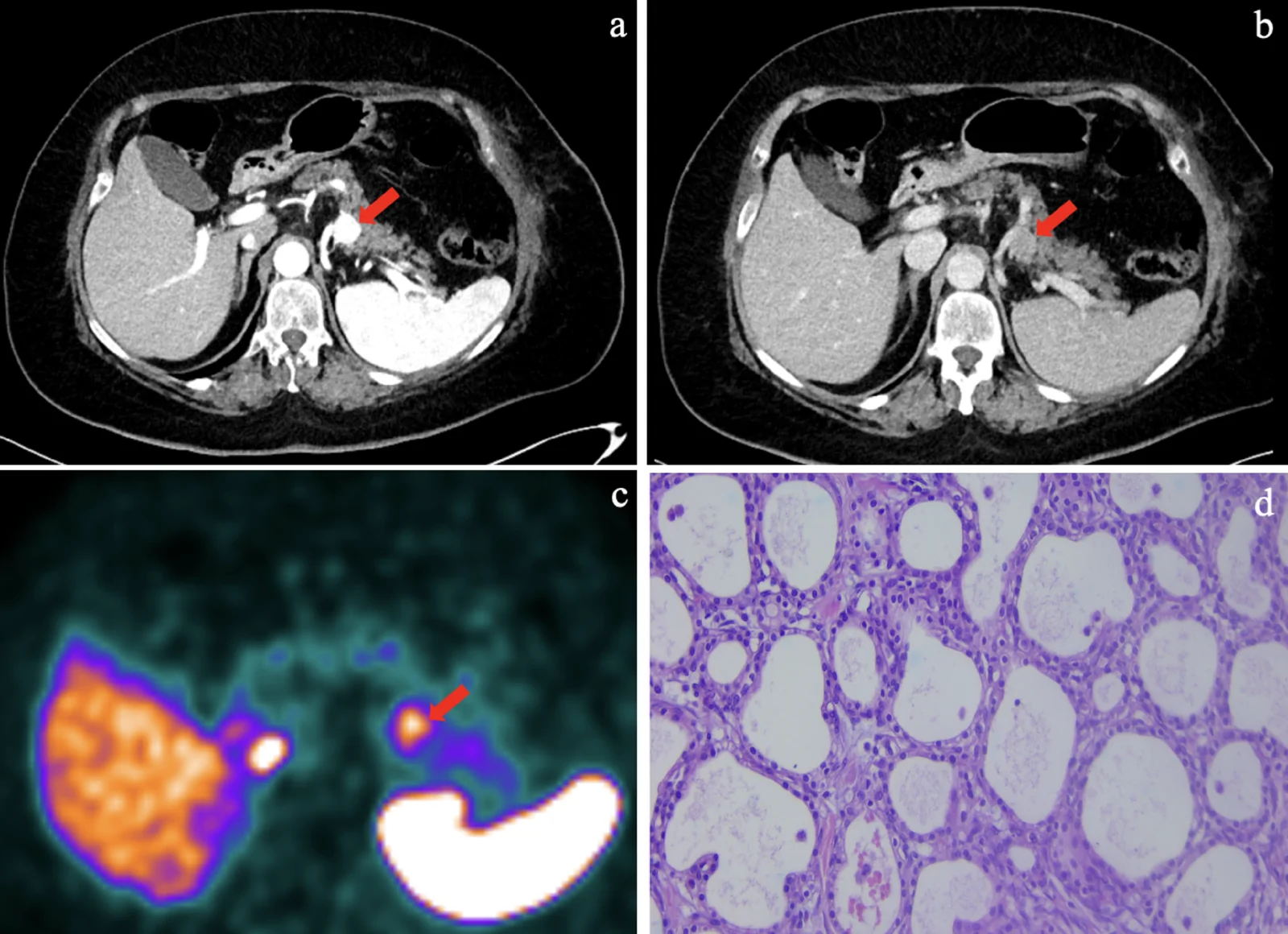
Illustrative case of microcystic serous cystadenoma mimicking a nonfunctioning pancreatic neuroendocrine tumour. **a** Arterial-phase contrast-enhanced CT demonstrating a well-circumscribed hyperenhancing lesion in the pancreatic tail (arrow), radiologically suggestive of NF-pNET. **b** Delayed venous-phase CT of the same lesion showing relative washout compared with the arterial phase (arrow). **c** ^68^Ga-DOTATATE PET/CT demonstrating avid tracer uptake within the lesion (SUVmax 9.8) (arrow). **d** Hematoxylin and eosin showing microcystic architecture consistent with serous cystadenoma. EUS-guided tissue acquisition was non-diagnostic; robot-assisted distal pancreatectomy with splenectomy confirmed the diagnosis

In our institutional experience, we recently managed an illustrative case in which arterial-phase contrast-enhanced CT and contrast-enhanced EUS strongly suggested NF-pNET. ^68^Ga-DOTATATE PET/CT demonstrated avid uptake within the lesion (SUVmax 9.8), whereas EUS-guided tissue acquisition was non-diagnostic. Given persistent diagnostic uncertainty, the patient underwent robot-assisted distal pancreatectomy with splenectomy, and final histopathology demonstrated microcystic SCA. Representative images are shown in Fig. 2.

EUS may still be valuable in selected cases, but evidence specific to this pitfall remains limited. The principal challenge is sampling: SCA are often paucicellular, and EUS-FNA frequently yields non-diagnostic material, even in solid-appearing variants. In the only comparative cohort reporting EUS-FNA performance among the included studies, diagnostic cytology was obtained in 0/5 SCA versus 6/10 NF-pNET cases. Case-based experience suggests that EUS-guided core biopsy (FNB), combined with cell-block preparation and immunohistochemistry, may secure a diagnosis in selected pseudo-solid microcystic SCA [48, 51], although diagnostic yield remains inconsistent. Therefore, a negative or non-diagnostic EUS sample should not be interpreted as excluding SCA. Rather, EUS-guided sampling should be considered when tissue confirmation would alter management, prioritizing core acquisition and an appropriate immunohistochemical panel.

The clinical relevance of this diagnostic pitfall lies in the operative pathway it may trigger. In the published misclassified cases, surgery frequently consisted of pancreatoduodenectomy or distal pancreatectomy, often with splenectomy in distal resections, indicating that benign lesions were not merely exposed to surgery, but to procedures of substantial magnitude. At the same time, postoperative events were incompletely and selectively reported, and the absence of clearly documented Clavien–Dindo grade ≥ IIIa morbidity should not be overinterpreted as evidence of low surgical burden. Rather, the available data suggest that the postoperative burden of misclassification is likely underestimated. This issue cannot be clarified by the comparative cohorts, because they included only resected patients and did not report operative or postoperative outcomes separately for SCA and NF-pNET.

**Fig. 3:**
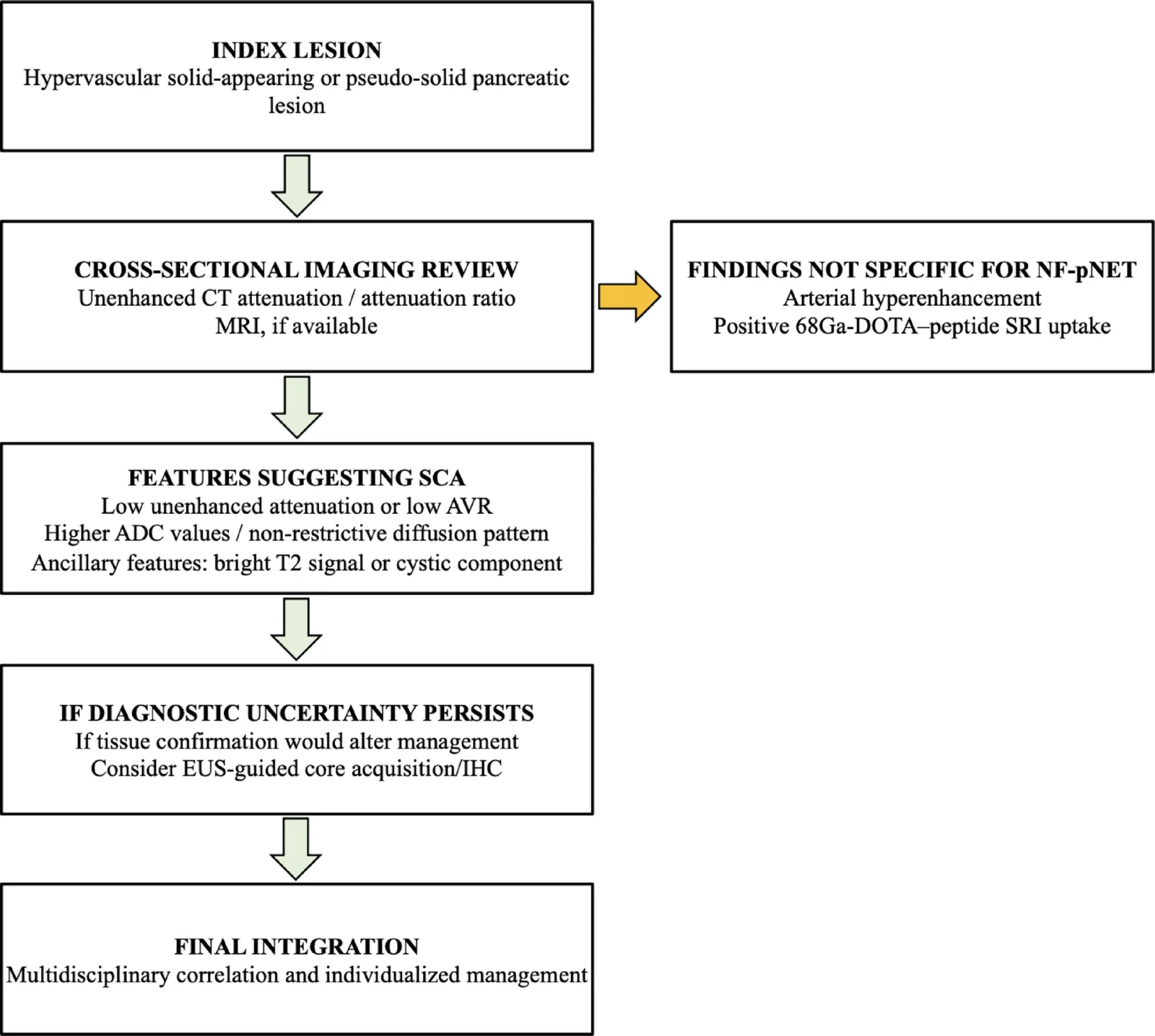
Suggested approach to selected hypervascular solid pancreatic lesions with possible SCA in the differential diagnosis. ADC, apparent diffusion coefficient; AVR, attenuation value ratio; CT, computed tomography; EUS, endoscopic ultrasonography; IHC, immunohistochemistry; MRI, magnetic resonance imaging; NF-pNET, nonfunctioning pancreatic neuroendocrine tumor; SCA, serous cystadenoma; SRI, somatostatin receptor imaging

Several limitations warrant emphasis. Comparative cohorts were retrospective resected series with high selection bias, and case-based publications were susceptible to publication and reporting bias. No externally validated diagnostic thresholds are currently available, and lack of standardized imaging protocols further reduces generalizability. These issues, together with non-overlapping quantitative metrics and incomplete technical reporting, also precluded meta-analysis. Moreover, most comparative data were generated before widespread integration of contemporary ^68^Ga-DOTA–peptide PET and current guideline-based diagnostic pathways. Accordingly, the present evidence is insufficient to support a definitive diagnostic algorithm or universal numeric cutoffs. Future studies should externally validate quantitative CT and MRI markers in broader, non-selected diagnostic populations and clarify how these features should be integrated with SRI and tissue sampling within multidisciplinary decision-making. Although the current evidence is insufficient to support a validated diagnostic algorithm, a schematic non-prescriptive framework derived from the available findings is provided in Fig. 3.

Taken together, the available evidence supports a pragmatic multiparametric approach rather than reliance on any single feature. In a hypervascular solid or pseudo-solid pancreatic lesion, low unenhanced attenuation and/or low attenuation ratios on CT and higher ADC values on MRI may raise suspicion for SCA, whereas arterial hyperenhancement or positive ^68^Ga-DOTA–peptide uptake alone should not be considered specific for NF-pNET. When imaging findings remain discordant and tissue confirmation would alter management, EUS-guided sampling—preferably with core acquisition and an appropriate immunohistochemical panel—may be considered within multidisciplinary review.

## Conclusions

NF-pNET–mimicking SCA is an uncommon but clinically consequential benign mimic that may appear solid or pseudo-solid and may also show somatostatin receptor imaging avidity. In the available retrospective comparative cohorts of resected, pathology-proven lesions, lower unenhanced CT attenuation (and attenuation ratios) and higher DWI–ADC values consistently favored SCA over NF-pNET, whereas arterial hyperenhancement and positive ^68^Ga-DOTA–peptide uptake were not specific. Accordingly, in hypervascular solid-appearing pancreatic lesions with discordant features, these quantitative CT/MRI markers may serve as useful adjuncts within multidisciplinary assessment, while EUS-guided tissue acquisition may be considered when tissue confirmation would alter management. Because current evidence derives from small, selected retrospective series without external validation, these findings should be applied cautiously and not as definitive diagnostic thresholds.

## Supplementary Information

Below is the link to the electronic supplementary material.

Supplementary Material 1.

## Data Availability

No new datasets were generated during the current study. The data supporting the findings of this study are derived from previously published articles, all of which are cited in the manuscript.
