# Supplementary material for: Hypervascular solid-appearing serous cystadenoma as a mimic of nonfunctioning pancreatic neuroendocrine tumour: a systematic review
Source: Abdom Radiol (NY). 2026 Apr 25;51(11):5644–55. doi: 10.1007/s00261-026-05532-y (PMC13672579; doi:10.1007/s00261-026-05532-y)
Supplement: Supplementary file 1 [file 261_2026_5532_MOESM1_ESM.docx]

**Table S1. Comparative cohort studies evaluating serous cystadenoma (SCA) vs nonfunctioning pancreatic neuroendocrine tumour (NF-pNET).**

| **Study (Author, year)** | **Study design** | **Population** | **Sample size (SCA / NF-pNET)** | **Lesion type** | **Imaging modalities** | **Reference standard** | **Key findings** |
| --- | --- | --- | --- | --- | --- | --- | --- |
| **Hayashi et al., 2012** | Retrospective comparative cohort | Surgically resected pancreatic hypervascular solid lesions | 3 / 15 | Solid serous cystadenoma vs pNET (NF-pNET subset included) | CT | Histopathology | SCA showed lower unenhanced and delayed attenuation, higher washout rate, and more frequent fibrous capsule |
| **Jang et al., 2015** | Retrospective comparative cohort | Surgically resected pancreatic hypervascular solid lesions | 10 / 15 | Solid-type SCA vs NF-pNET | CT; MRI | Histopathology | MRI-DWI outperformed CT; SCA showed lower unenhanced attenuation and higher ADC |
| **Park et al., 2016** | Retrospective comparative cohort | Surgically resected hypervascular solid-appearing pancreatic lesions | 15 / 30 | Hypervascular solid-appearing SCA vs NF-pNET | CT; MRI | Histopathology | SCA showed lower unenhanced attenuation, bright T2 signal, cystic component >10%, and non-restrictive ADC |
| **Fang et al., 2022** | Retrospective comparative cohort (PSM) | Surgically resected pancreatic solid lesions | 22 / 29 | Solid-type SCA vs NF-pNET | CT | Histopathology | Low unenhanced attenuation value ratio (AVR) and wash-in/wash-out pattern favoured SCA |

Abbreviations: ADC, apparent diffusion coefficient; AVR, attenuation value ratio; CT, computed tomography; DWI, diffusion-weighted imaging; MRI, magnetic resonance imaging; NF-pNET, nonfunctioning pancreatic neuroendocrine tumour; pNET, pancreatic neuroendocrine tumour; PSM, propensity score matching; SCA, serous cystadenoma.

**Table S2. Case-based studies reporting serous cystadenoma (SCA) mimicking nonfunctioning pancreatic neuroendocrine tumours (NF-pNET).**

| **Study (Author, year)** | **Study type** | **No. of patients** | **Imaging modalities** | **SRI** | **Management** | **Key diagnostic pitfall** |
| --- | --- | --- | --- | --- | --- | --- |
| **Yamamoto et al., 2004** | Case report | 1 | US; CT; MRI; MRCP; angiography | Not reported | Enucleation | Uniform arterial hyperenhancement |
| **Gabata et al., 2005** | Case report | 1 | CT; MRI; MRCP; angiography | Not reported | DP | Hypervascular solid mass on CT |
| **Yamaguchi et al., 2006** | Case report (letter) | 1 | CT; ultrasonography | Not reported | DP | Well-enhanced solid mass with ductal dilatation |
| **Reese et al., 2006** | Case report | 1 | CT | Not reported | PD | Large hypervascular solid lesion |
| **Matsumoto et al., 2006** | Case report | 1 | US; dynamic CT | Not reported | Enucleation | Strong homogeneous arterial enhancement |
| **Sanaka et al., 2007** | Case report | 1 | CT; EUS | Not reported | Enucleation ± pancreaticojejunostomy | Hypervascular solid mass; inconclusive FNA |
| **Stern et al., 2007** | Case report | 1 | CT | Not reported | DP + splenectomy | Solid hypervascular mass on CT |
| **Casadei et al., 2008** | Case report | 1 | CT; US | Not reported | DP + splenectomy | Solid hypervascular lesion |
| **Lam-Himlin et al., 2010** | Case report | 1 | CT | Not reported | DP + splenectomy | Solid hypervascular lesion |
| **Yasuda et al., 2011** | Case report | 1 | CT; MRI | Not reported | PD | Solid hypervascular lesion |
| **Lee et al., 2013** | Case report | 1 | CT | Not reported | DP | Persistently enhancing solid mass |
| **Kishida et al., 2014** | Case report | 1 | US; CT; MRI; MRCP; EUS (Doppler) | Not reported | DP | Peripheral hypervascular enhancement |
| **Wu et al., 2015** | Case series | 2 | CT | Positive (Octreoscan in 1/2) | PD (1); DP (1) | Arterial hyperenhancement with positive SRI |
| **Geramizadeh et al., 2016** | Case report | 1 | CT | Not reported | PD | Well-defined hyperenhancing mass |
| **Katsourakis et al., 2016** | Case report | 1 | MRI | Not reported | DP | Arterial enhancement with wash-out |
| **Hamid et al., 2017** | Case report | 1 | US; CT | Not reported | DP + splenectomy | Solid mass with moderate enhancement |
| **Chen et al., 2019** | Case report | 1 | CT; MRI; MRCP | Not reported | Surgical resection (not specified) | Solid hypervascular enhancement |
| **Demesmaker et al., 2019** | Case report | 1 | CT; MRI; EUS | Negative | PD + liver resections | Solid hypervascular lesion |
| **Manfrin et al., 2019** | Case series | 7 | CT; MRI; EUS (Doppler) | Negative (⁶⁸Ga-PET) | Surveillance | Pseudo-solid hypervascular appearance |
| **Nunes et al., 2020** | Case series | 2 | CT; EUS (Doppler) | Negative | PD (1); surveillance (1) | Solid hypervascular lesion |
| **Nappo et al., 2020** | Case report | 1 | CT; MRI; EUS; PET/CT | Positive (⁶⁸Ga-DOTATOC) | DP + splenectomy | Hypervascular solid-appearing lesion with positive SRI |
| **Bringeland et al., 2022** | Case report | 1 | CT, PET/CT | Positive (⁶⁸Ga-DOTATOC) | DP | Hypervascular peripheral rim with positive SRI |
| **Nakamura et al., 2022** | Case report | 1 | CT; MRI; MRCP; EUS | Not reported | Spleen-preserving DP | Small hypervascular solid-appearing lesion |
| **Saragoni et al., 2022** | Case report | 1 | MRI; CE-EUS; PET/CT | Positive (⁶⁸Ga-DOTANOC) | DP + splenectomy | Hypervascular solid lesion with positive SRI |
| **Passand et al., 2022** | Case report | 1 | CT; MRI; EUS; PET/CT | Positive (⁶⁸Ga-DOTATOC) | PD | Cystic lesion with positive SRI |
| **Canakis et al., 2022** | Case report | 1 | EUS; SRI | Positive (Octreoscan) | Surveillance | Positive SRI |
| **Chen et al., 2023** | Case report | 1 | CT; PET/CT | Positive (⁶⁸Ga-DOTATATE) | PD | Hypervascular solid lesion with positive SRI |
| **Gao et al., 2023** | Case report | 1 | CT; PET/MRI | Positive (⁶⁸Ga-DOTATATE) | PD | Hypervascular lesion with positive SRI |
| **Cai et al., 2024** | Case report | 1 | CT; SPECT/CT | Positive (99mTc-HYNIC-TOC) | PD | Hypervascular lesion with positive SRI |
| **Kumarasamy et al., 2023** | Case report | 1 | CT, PET/CT | Positive (⁶⁸Ga-DOTATATE) | PD | Enhancing solid mass with positive SRI |
| **da Costa et al., 2025** | Case report | 1 | MRI | Not performed | Laparoscopic PD | Marked arterial hyperenhancement |

Abbreviations: CE-EUS, contrast-enhanced endoscopic ultrasonography; CT, computed tomography; DP, distal pancreatectomy; EUS, endoscopic ultrasonography; FNA, fine needle aspiration; MRCP, magnetic resonance cholangiopancreatography; MRI, magnetic resonance imaging; NF-pNET, nonfunctioning pancreatic neuroendocrine tumour; PD, pancreatoduodenectomy; PET/CT, positron emission tomography/computed tomography; SCA, serous cystadenoma; SPECT/CT, single-photon emission computed tomography/computed tomography; SRI, somatostatin receptor imaging; US, ultrasonography.

**Table S3. QUADAS-2 assessment of comparative cohort studies.**

| **Study (Author, year)** | **RoB: Patient selection** | **RoB: Index test** | **RoB: Reference standard** | **RoB: Flow & timing** | **Applic.: Patient selection** | **Applic.: Index test** | **Applic.: Reference standard** |
| --- | --- | --- | --- | --- | --- | --- | --- |
| **Hayashi et al., 2012** | High | Unclear | Low | High | High | Unclear | Low |
| **Jang et al., 2015** | High | Unclear | Low | Low | High | Low | Low |
| **Park et al., 2016** | High | High | Low | Unclear | High | Low | Low |
| **Fang et al., 2022** | High | High | Low | High | High | Low | Low |

Abbreviations: RoB, risk of bias; SCA, serous cystadenoma; NF-pNET, nonfunctioning pancreatic neuroendocrine tumour.

Legend: green = low; red = high; yellow = unclear.

**Table S4. Risk of bias assessment of case reports and case series (Murad et al.).**

| **Study (Author, year)** | **Design (n)** | **Reference standard** | **Imaging Modalities** | **1 Selection** | **2 Exposure ascertainment** | **3 Outcome ascertainment** | **4 Alternative causes addressed** | **7 Follow-up** | **8 Reporting** | **Overall judgement** |
| --- | --- | --- | --- | --- | --- | --- | --- | --- | --- | --- |
| **Yamamoto et al., 2004** | Case report (1) | Surgical histology | CT; MRI | No | Yes | Yes | Yes | No | Yes | **Moderate** |
| **Yamaguchi et al., 2006** | Case report (1) | Surgical histology | CT | No | Yes | Yes | Yes | Yes | Yes | **Low** |
| **Gabata et al., 2005** | Case report (1) | Surgical histology | CT; MRI | No | Yes | Yes | Yes | No | Yes | **Moderate** |
| **Matsumoto et al., 2006** | Case report (1) | Surgical histology | CT | No | Yes | Yes | Yes | Yes | Yes | **Low** |
| **Reese et al., 2006** | Case report (1) | Surgical histology | CT | No | Yes | Yes | Yes | Yes | Yes | **Moderate** |
| **Sanaka et al., 2007** | Case report (1) | Surgical histology | CT; EUS | No | Yes | Yes | Yes | Yes | Yes | **Moderate** |
| **Stern et al., 2007** | Case report (1) | Surgical histology | CT | No | Yes | Yes | Yes | No | Yes | **Moderate** |
| **Casadei et al., 2008** | Case report (1) | Surgical histology | CT | No | Yes | Yes | Yes | Yes | Yes | **Moderate** |
| **Lam-Himlin et al., 2010** | Embedded case in review (1) | Surgical histology | CT | No | Yes | Yes | Yes | Yes | Yes | **Moderate** |
| **Yasuda et al., 2011** | Case report (1) | Surgical histology | CT; MRI; EUS | No | Yes | Yes | Yes | No | Yes | **Moderate** |
| **Lee et al., 2013** | Case report (1) | Surgical histology | CT | No | Yes | Yes | Yes | Yes | Yes | **Moderate** |
| **Kishida et al., 2014** | Case report (1) | Surgical histology | CT; MRI | No | Yes | Yes | Yes | Yes | Yes | **Low** |
| **Wu et al., 2015** | Case series (2) | Surgical histology (2/2) | CT; SRI | Unclear | Yes | Yes | Yes | Unclear | Yes | **Moderate** |
| **Katsourakis et al., 2016** | Case report (1) | Surgical histology | MRI | No | Yes | Yes | Yes | Yes | Yes | **Moderate** |
| **Geramizadeh et al., 2016** | Case report (1) | Surgical histology | CT | No | Yes | Yes | Yes | Yes | Yes | **Moderate** |
| **Hamid et al., 2017** | Case report (1) | Surgical histology | CT | No | Yes | Yes | Yes | Yes | Yes | **Moderate** |
| **Demesmaker et al., 2019** | Case report (1) | Surgical histology | CT; MRI; SRI; EUS | No | Yes | Yes | Yes | Yes | Yes | **Moderate** |
| **Chen et al., 2019** | Case report (1)** | Surgical histology | CT; MRI; MRCP | No | Yes | Yes | Yes | No | Yes | **Moderate** |
| **Manfrin et al., 2019** | Case series (7) | Core biopsy histology (7/7) | CT; MRI; SRI; EUS | Yes | Yes | Yes* | Yes | Yes | Yes | **Moderate** |
| **Nunes et al., 2020** | Case series (2) | Mixed: surgical histology (1/2); core biopsy histology (1/2) | CT; EUS; SRI | Unclear | Yes | Unclear | Yes | Unclear | Yes | **Moderate** |
| **Nappo et al., 2020** | Case report (1) | Surgical histology | CT; MRI; SRI; EUS | No | Yes | Yes | Yes | No | Yes | **Moderate** |
| **Bringeland et al., 2022** | Case report (1) | Surgical histology | CT; SRI | No | Yes | Yes | Yes | No | Yes | **Moderate** |
| **Nakamura et al., 2022** | Case report (1) | Surgical histology | CT; MRI; EUS | No | Yes | Yes | Yes | No | Yes | **Moderate** |
| **Passand et al., 2022** | Case report (1) | Surgical histology | CT; MRI; SRI; EUS | No | Yes | Yes | Yes | No | Yes | **Moderate** |
| **Canakis et al., 2022** | Case report (1) | Cytology only (EUS-FNA) | SRI; EUS | No | Yes | Unclear | Unclear | No | Yes | **High** |
| **Saragoni et al., 2022** | Case report (1) | Surgical histology | MRI; SRI; EUS | No | Yes | Yes | Yes | No | Yes | **Moderate** |
| **Chen et al., 2023** | Case report (1) | Surgical histology | CT; SRI | No | Yes | Yes | Yes | No | Yes | **Moderate** |
| **Gao et al., 2023** | Case report (1) | Surgical histology | CT; MRI; SRI | No | Yes | Yes | Yes | No | Yes | **Moderate** |
| **Kumarasamy et al., 2023** | Case report (1) | Surgical histology | CT; SRI | No | Yes | Yes | Yes | Yes | Yes | **Moderate** |
| **Cai et al., 2024** | Case report (1) | Surgical histology | CT; SRI | No | Yes | Yes | Yes | No | Yes | **Moderate** |
| **da Costa et al., 2025** | Case report (1) | Surgical histology | MRI | No | Yes | Yes | Yes | Yes | Yes | **Low** |

Abbreviations: CT, computed tomography; EUS, endoscopic ultrasonography; EUS-FNA, endoscopic ultrasound-guided fine-needle aspiration; MRCP, magnetic resonance cholangiopancreatography; MRI, magnetic resonance imaging; NF-pNET, nonfunctioning pancreatic neuroendocrine tumour; SCA, serous cystadenoma; SRI, somatostatin receptor imaging.

Items 5 (challenge/rechallenge) and 6 (dose-response) were not applicable to diagnostic imaging case reports/series and were recorded as N/A. Overall judgements were used to inform interpretation but did not determine study inclusion.

* Outcome ascertainment in Manfrin 2019 was based on core biopsy histology with immunohistochemistry; no surgical reference standard was available.

** In Chen et al., 1/5 cases was eligible, as NF-pNET was explicitly considered in the differential diagnosis.

**Table S5. Technical imaging parameters, measurement methods, and reported diagnostic thresholds in comparative cohort studies evaluating serous cystadenoma versus NF-pNET.**

| **Study (Author, year)** | **Study design and cohort** | **CT acquisition protocol** | **MRI/DWI protocol** | **Measurement method** | **Main quantitative metrics / reported thresholds** | **Methodological considerations relevant to interpretation** |
| --- | --- | --- | --- | --- | --- | --- |
| **Hayashi et al., 2012** | Retrospective single-center study; 3 solid serous cystadenomas vs 15 endocrine tumours, all surgically resected | Two CT systems (4-row and 64-row) and 3 different protocols used over time. Protocol 1: unenhanced, early (40 s), and delayed (180 s). Protocol 2: unenhanced, early (40 s), and delayed (180 s), with contrast dose 600 mgI/kg. Protocol 3: unenhanced, arterial (40 s), portal (70 s), and delayed (240 s). Reconstruction parameters differed between 4-row and 64-row CT platforms | Not applicable | When digital data were available, a circular ROI covering the whole lesion was used to record CT attenuation by phase. Relative washout rate (RWR) and absolute washout rate (AWR) were calculated from unenhanced, early, and delayed phase values | Mean unenhanced attenuation: 28.0 ± 8.5 HU in SSCA vs 48.4 ± 11.2 HU in ET. Mean early-phase attenuation: 188.5 ± 77.0 HU in SSCA vs 181.1 ± 55.0 HU in ET. Mean delayed attenuation: 82.5 ± 27.6 HU in SSCA vs 126.2 ± 35.3 HU in ET. RWR: 0.55 ± 0.04 in SSCA vs 0.29 ± 0.21 in ET. No ROC-derived numeric cutoff was reported | Very small SSCA sample; marked heterogeneity of CT protocols over a long accrual period; incomplete digital data availability; no MRI/DWI data; no externally usable ROC threshold |
| **Jang et al., 2015** | Retrospective single-center study; 10 solid-type SCNs vs 15 surgically resected NETs; all lesions 10–30 mm and hypervascular on preoperative CT/MRI | CT performed with either pancreas protocol or liver protocol on 4 multidetector scanners. Pancreas protocol: unenhanced, arterial, pancreatic, and portal phases. Liver protocol: unenhanced, arterial, portal, and equilibrium phases. 120 kVp; 189–200 mAs; section thickness about 3 mm. Contrast: 120 mL iodinated contrast at 3–4 mL/s | All patients underwent 3-T MRI with gadoxetic acid-enhanced imaging, MRCP, and respiratory-triggered DWI. DWI b values: 0, 100, and 800 s/mm². ADC calculated using b values of 100 and 800 s/mm² | Circular or ovoid ROIs placed in the corresponding solid hypervascular-enhancing portion of the tumour on non-enhanced CT and ADC map; measurements repeated 3 times and averaged. Mean lesion ROI area: 42.6 ± 21.95 mm² | Mean non-enhanced attenuation: 18.5 ± 8.16 HU in SCN vs 39.2 ± 7.76 HU in NET. Mean ADC: 2.45 ± 0.27 × 10⁻³ mm²/s in SCN vs 1.51 ± 0.46 × 10⁻³ mm²/s in NET. ROC-derived cutoffs: <32 HU for non-enhanced CT and >1.99 × 10⁻³ mm²/s for ADC | CT protocols were heterogeneous across patients; MRI and DWI were standardized but ROI analysis targeted the solid hypervascular portion rather than the whole lesion; retrospective resected cohort |
| **Park et al., 2016** | Retrospective single-center study; 15 hypervascular solid-appearing SCNs vs 30 size-matched surgically proven NETs | Multidetector CT using 4 scanner types; non-enhanced, arterial, and portal venous phases; 120 kVp; automated dose modulation with maximum tube current 200 mAs; beam collimation 0.625–1.5 mm; reconstruction thickness 3 mm. Contrast-enhanced CT performed with iopromide (2 mL/kg at 3 mL/s); arterial phase triggered by bolus tracking at 100 HU in the upper abdominal aorta with 10-s diagnostic delay; portal phase at 72 s after contrast injection | Preoperative MRI available in 5 SCN and 17 NET patients; 1.5-T scanner; dynamic contrast-enhanced MRI plus DWI. DWI acquired with 4 b values (0, 150, 500, and 1000 s/mm²) | CT attenuation values measured in HU using ROI placement; 3 measurements at different levels were averaged. ADC values also measured 3 times at different levels and averaged | Mean non-enhanced attenuation: 29.6 ± 9.5 HU in SCN vs 38.8 ± 9.9 HU in NET. Mean ADC: 1.621 ± 0.139 × 10⁻³ mm²/s in SCN vs 1.056 ± 0.303 × 10⁻³ mm²/s in NET. ROC-derived cutoffs: <34 HU for non-enhanced CT and >1.36 × 10⁻³ mm²/s for ADC | Only a subset underwent MRI; CT performed on multiple scanner platforms; analysis restricted to hypervascular solid-appearing SCNs and size-matched NETs; retrospective resected cohort |
| **Fang et al., 2022** | Retrospective single-center cross-sectional study; 22 solid SCAs and 120 NF-pNETs, all surgically confirmed; propensity score matching yielded 22 SCAs vs 29 NF-pNETs for matched analysis | CT performed on 320-slice or 256-slice multidetector scanners; non-enhanced plus triple-phase contrast-enhanced CT. Tube voltage 120 kV, tube current 250 mA, rotation time 0.5 s. Test bolus method used. Contrast: 90–95 mL iopromide 370 mgI/mL at 4–5 mL/s followed by 20 mL saline flush. Arterial, portal venous, and delayed phases acquired at 20–25 s, 60–70 s, and 110–130 s, respectively. Slice thickness/reconstruction interval 0.8/1.0 mm and 1.0/1.0 mm | Not applicable | Largest possible spherical ROI placed in the tumour and in unaffected normal pancreas in each phase, excluding pancreatic duct and partial-volume contamination; ROI attenuation measured 3 times and averaged. AVR defined as tumour attenuation / normal pancreas attenuation | Unenhanced AVR: 0.59 ± 0.12 in SCA vs 0.92 ± 0.13 in NF-pNET. Arterial AVR: 1.10 ± 0.42 vs 1.49 ± 0.60. Portal venous AVR: 0.90 ± 0.25 vs 1.23 ± 0.31. Delayed AVR: 0.90 ± 0.23 vs 1.22 ± 0.22. Best-performing cutoff: unenhanced AVR <0.77. Additional phase-specific cutoffs: arterial 1.08, portal venous 0.99, delayed 0.95. Enhancement pattern was also evaluated | CT-only study; attenuation normalized to pancreatic parenchyma using AVR rather than absolute HU; matched analysis after PSM; retrospective resected surgical cohort |

Abbreviations: ADC, apparent diffusion coefficient; AVR, attenuation value ratio; DWI, diffusion-weighted imaging; ET, endocrine tumour; HU, Hounsfield unit; MR, magnetic resonance; MRCP, magnetic resonance cholangiopancreatography; NF-pNET, nonfunctioning pancreatic neuroendocrine tumour; ROI, region of interest; RWR, relative washout rate; SCA/SCN, serous cystadenoma/serous cystic neoplasm; SSCA, solid serous cystadenoma.
